# Marine phage genomics: the tip of the iceberg

**DOI:** 10.1093/femsle/fnw158

**Published:** 2016-06-22

**Authors:** Blanca Perez Sepulveda, Tamsin Redgwell, Branko Rihtman, Frances Pitt, David J. Scanlan, Andrew Millard

**Affiliations:** 1 School of Life Sciences, University of Warwick, Gibbet Hill Road, Coventry, CV4 7AL, UK; 2 Warwick Medical School, University of Warwick, Gibbet Hill Road, Coventry, CV4 7AL, UK

**Keywords:** bacteriophage, genomics, marine

## Abstract

Marine viruses are the most abundant biological entity in the oceans, the majority of which infect bacteria and are known as bacteriophages. Yet, the bulk of bacteriophages form part of the vast uncultured dark matter of the microbial biosphere. In spite of the paucity of cultured marine bacteriophages, it is known that marine bacteriophages have major impacts on microbial population structure and the biogeochemical cycling of key elements. Despite the ecological relevance of marine bacteriophages, there are relatively few isolates with complete genome sequences. This minireview focuses on knowledge gathered from these genomes put in the context of viral metagenomic data and highlights key advances in the field, particularly focusing on genome structure and auxiliary metabolic genes.

## Abstract (precis)

Only a tiny fraction of marine phages have been discovered, yet are known to have important roles in the ocean.

## INTRODUCTION

**Figure 1.**
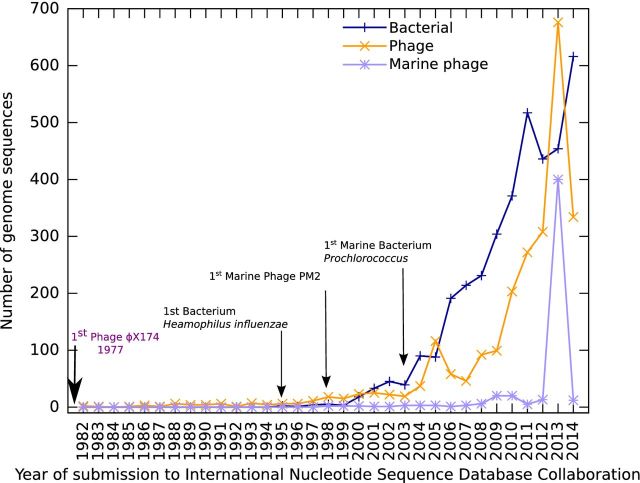
Number of bacterial, phage and marine phage genomes submitted per year to the International Nucleotide Sequence Database Collaboration (INSDC). Dates were extracted for all sequences within the EBI phage database (http://www.ebi.ac.uk/genomes/phage.html). Data are only shown from 1982 onwards with the inception of publically available databases. Dates are representative of when the sequence was submitted to INSDC rather than when any subsequent papers were published. Prophages are not included unless they have been specifically sequenced independently of their host bacterium. Phages were classified as marine if they were isolated from a marine environment.

Marine viruses are found at concentrations up to ∼1 × 10^8^ ml^−1^, resulting in an estimated ∼4 × 10^30^ viruses in the oceans (Suttle 2005). They play a key role in the biogeochemical cycling of major elements, for example by diverting the flow of carbon into dissolved and particulate organic matter through the lysis of their bacterial hosts, thus influencing the amount of carbon that is sequestered to the deep ocean by the biological pump (see Suttle 2007 for review). Despite the importance of marine bacteriophages, there are relatively few isolates for which complete genome sequences are available (from a total of 2010 bacteriophage genome sequences 482 belong to marine bacteriophages; Fig. 1); therefore, the majority of bacteriophages form part of the vast uncultured dark matter of the microbial world.

It is now over 30 years since the genome of the 5.3-kb ssDNA bacteriophage φX174 was completed (Sanger *et al.*^1977^), leading to the sequencing of the first dsDNA genomes of bacteriophages lambda and T7 (Sanger *et al.*^1982^; Dunn and Studier 1983). These smaller bacteriophage (hereafter phage) genomes were completed many years before the first bacterial genome was published in 1995 (Fleischmann *et al.*^1995^; Fig. 1). The first marine phage genome, PM2, infecting the heterotroph *Pseudoalteromonas*, was completed over two decades after sequencing of φX174 (Mannisto *et al.*^1999^), with the first marine phage infecting a photoautrophic host (*Synechococcus*) not completed until a few years later (Chen and Lu 2002; Fig. 1).

The number of bacterial and phage genomes has increased dramatically in the last 10 years, with 10 times as many phage genomes sequenced post 2004 than in the preceding two decades, in line with the decreasing per-base cost of sequencing (Fig. 1). Notably, the number of phage genomes deposited in 2013 exceeds that of bacterial genomes (Fig. 1). However, there are more assembled bacterial (3316) than phage genomes (2010) within the European Nucleotide Archive (ENA: http://www.ebi.ac.uk/genomes/; Fig. 1). This is because despite the early sequencing efforts in phage research and their smaller genomes relative to bacteria, thousands of bacterial isolates are being sequenced (Eyre *et al.*^2013^; Chewapreecha *et al.*^2014^; Nasser *et al.*^2014^) and the data submitted into whole genome sequencing databases as raw read data (https://www.ebi.ac.uk/genomes/wgs.html), without assembly of the genome. To date there are no studies carried out at the same scale for phages.

This is even more pronounced for marine phages; whilst the number of deposited phage genomes increased sharply post 2004, this did not occur for marine phages (Fig. 1). There was a substantial increase in 2013 as a result of the seminal work by Mizuno *et al.* (2013a,b), which assembled 208 complete marine phage genomes from metagenomic samples and the further submission of 139 *Synechococcus* phage isolates. However, the submissions in 2013 are an anomaly from the general trend as only 11 marine phage genomes were submitted in 2014. Thus, despite the ability to readily isolate marine phages and technological advances in sequencing, the number of marine phage genomes submitted is similar to that of levels 10 years previously. This observation suggests that the area of marine phage genomics has yet to fully utilise the potential of high-throughput sequencing.

**Figure 2.**
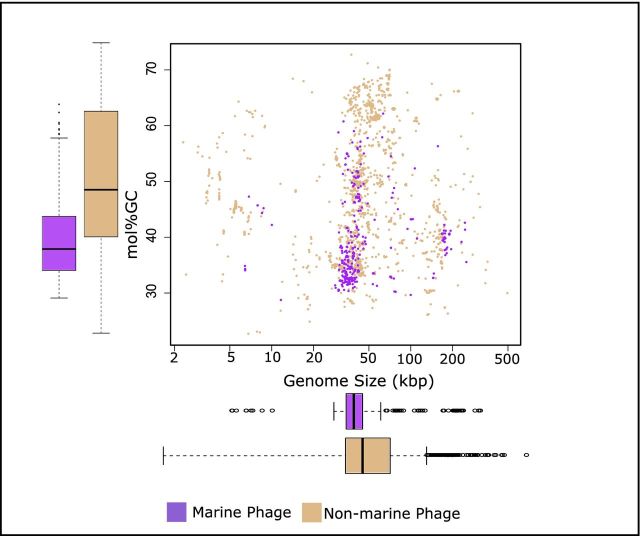
Scatter plot of genome size versus mol %G + C of marine phages and non-marine phages. Box and whisper plots for the range in genome size and range in mol %G + C are also plotted adjacent to the *Y-* and *X*-axis, respectively.

The relatively small number and diversity of marine phages can also be deduced by examining the broad genome parameters of the sequences deposited in the ENA. A comparison of molar G + C content (mol%GC) and genome size revealed a limited range of these parameters for marine phages (Fig. 2). Whilst this may be a specific adaptation to the marine environment that selects for a certain mol%GC, it more likely results from the bias of isolation performed on a small number of hosts, not representing the true diversity of marine phages (Table S1, Supporting Information). In this regard, it is clearly noticeable that there are very few marine phage genomes which have a high mol%GC content (Fig. 2). However, the presence of marine bacteria with high mol%GC is well documented (Subramani and Aalbersberg 2012), highlighting even in the broad terms of genome size and mol%GC that there are large numbers of phages for which there are no sequenced representatives.

With an estimated 5476 pelagic viral populations in the world's oceans (Brum et al. 2015), only very few have cultured isolates and even less have representative genomes. Currently, there are 482 complete marine phage genomes within the ENA (May 2015), for 208 of which the host has not been cultured since they were assembled from metagenomic samples, and the remainder isolated from merely 22 genera of bacteria (Table S1, Supporting Information). With an estimated 2 × 10^6^ bacterial taxa present in the ocean (Curtis, Sloan and Scannell 2002), having representative phages isolated on just 22 bacterial genera highlights how much is still to be discovered. Despite sequencing only encompassing the tip of the phage iceberg, insights from these genomes have radically changed our understanding of genome evolution, phage-host interactions and phage ecology. This review will focus on what has been learnt from the genomes of cultured marine phage isolates, with a brief summary of the culture-independent methods of phage genome sequencing.

## CULTURE-DEPENDENT VERSUS CULTURE-INDEPENDENT GENOME SEQUENCING

Historically, phage genome sequencing has relied on the traditional culturing of a bacterial host; thus, the isolation and purification of phage is limited by the very need to culture the host bacterium. Furthermore, the construction of shotgun clone libraries from phage DNA presented technical difficulties, including the presence of modified nucleotides and possession of toxic genes in phage genomes (Breitbart *et al.*^2002^). Whilst host isolation remains a bottleneck, the advent of high-throughput sequencing methods has alleviated the problems associated with clone libraries. The relatively small size of phage genomes (phage median genome size 44.8 kb; bacterial median genome size 3.04 Mb) makes them ideal candidates for sequencing, with complete genome assembly possible for the majority (Rihtman *et al.*^2016^). The use of bench top sequencers offers the potential to sequence ∼200 phage genomes in a single run, with minimal method optimisation. Whilst isolation will continue to be an issue, there are numerous reports of the isolation of novel phages (Wichels *et al.*^1998^; Alonso, Rodríguez and Borrego 2002), but there is no genome information for these phages. Even for well-developed systems where the host bacteria can be cultured, there has been minimal utilisation of current sequencing technologies to sequence large numbers of phages. This suggests that current technologies are not being fully used to study the genomes of cultured marine phages.

To alleviate the issues of traditional culture-based methods, a number of metagenomic-based approaches have been used to study marine phage genomics. Marine phage genomics has been at the forefront of metagenomics since the pioneering work of Breitbart *et al.* (2002). Since then, there have been numerous shotgun metagenomic studies that have sequenced small fragments to explore viral diversity (e.g. see Angly *et al.*^2006^; Dinsdale *et al.*^2008^; Sharon *et al.*^2011^; McDaniel *et al.*^2014^) or constructed large fosmid libraries allowing the reconstruction of near-complete marine phage genomes (Mizuno *et al.*2013a,b). Whilst metagenomics has vastly increased our understanding of marine phage diversity, the initial drawback was that it prevented the unambiguous assignment of phage to their respective hosts. The advent of single-cell amplified genomes (Rodrigue *et al.*^2009^) has gone some way to resolve the issue of who infects whom, with phages identified within bacterial single-cell amplified genomes. For example, the recent analysis of SUP05 bacterial single-cell amplified genomes identified co-infection with both dsDNA and ssDNA phages, including contigs representing new genera within the *Caudovirales* and *Gokushoviriniae* families (Roux *et al.*^2014^). Furthermore, the latest combination of viral tagging (Deng *et al.*^2012^) with metagenomics is now able to link the genomes of environmental phage isolates and provide host information (Deng *et al.*^2014^). Recent advances in marine viral metagenomics are thoroughly reviewed elsewhere (Brum and Sullivan 2015). Despite all the pros of metagenomics, the lack of phage isolates still limits further experimental work critical for understanding the dynamics of phage–host interactions.

## SHEDDING LIGHT ON PHAGE DARK MATTER

Whilst the use of metagenomics has started to reveal the vast diversity of phage genomes, a common feature of these metagenomic datasets is that very few sequences can be assigned to known phage isolates, e.g. 87%–93% of the Pacific Ocean virome could not be associated to any viral taxa (Hurwitz and Sullivan 2013). Hence, sequencing of phage isolates can make substantial contributions to the analysis of these large metagenomic datasets. This is demonstrated by the sequencing of four phages infecting the ubiquitous SAR11 clade, the most abundant marine bacterial lineage, accounting for between 22% and 55% of prokaryotic cells (Morris *et al.*^2002^). The small cell size and slow growth of these bacteria, attributes that could potentially impede viral replication, initially led to the idea that these organisms were resistant to viral infection (Yooseph *et al.*^2010^). However, the recent isolation of four SAR11-infecting phages (pelagiphages) enabled determination of their abundance and distribution in metagenomic samples, with HTVC010P-like pelagiphages 2.5× more abundant than all *T4likeviruses* combined (Zhao *et al.*^2013^).

Shortly after the characterisation of the ubiquitous pelagiphage, phage HMO-2011 was isolated and described (Kang *et al.*^2013^). This phage infects *Candidatus Puniceispirillum marinum* strain IMCC1322, a representative of the SAR116 clade which contributes up to 10% of the bacterial assemblage in euphotic zone waters (Kang *et al.*^2013^). HMO-2011 usurped HTVC010P as the most abundant phage in marine viral metagenomic libraries, with up to 25% of taxonomically identifiable reads assigned to HMO-2011 (Kang *et al.*^2013^), once again demonstrating the power of studying individual phage isolates to understand the global distribution of marine phages.

Even for the most well studied of marine phage-host systems, sequencing of additional phage isolates can have a significant impact on identifying the viral dark matter, as exemplified by cyanophage S-TIM5 (Sabehi *et al.*^2012^). S-TIM5 lacks the structural module that is conserved between cyanophages and *T4likeviruses*, containing instead a novel set of structural and replication genes (Sabehi *et al.*^2012^). These include a mitochondrial-like DNA polymerase that is also found in a phage infecting *Acaryochloris* (Chan *et al.*^2015^), providing further support for the idea of a phage origin for mitochondrial DNA polymerase (Chan *et al.*^2011^, ^2015^; Sabehi *et al.*^2012^). Comparison of the S-TIM5 genome against metagenomic datasets demonstrated the widespread distribution of this previously unknown phage type within the environment (Sabehi *et al.*^2012^).

The vast viral metagenomic datasets that are available (Hurwitz and Sullivan 2013; Brum *et al.*^2015^) provide a powerful resource for determining the distribution of phage groups. However, far more individual phage isolates need to be sequenced so that the majority of metagenomic reads can be assigned to a phage taxa, rather than the minority.

## GENOME STRUCTURE AND EVOLUTION

**Figure 3.**
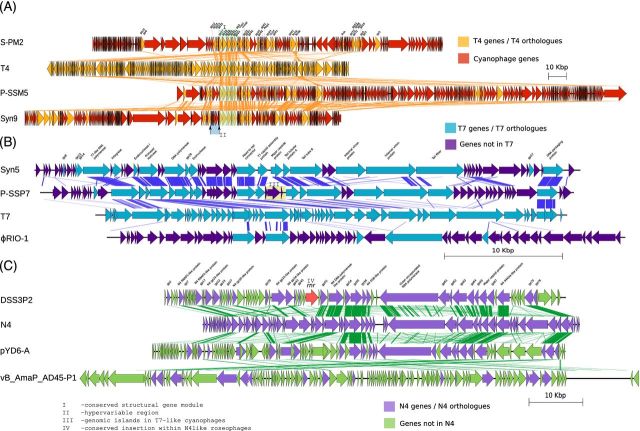
Genomic comparison of selected phages. (**A**) Cyanophage S-PM2, P-SSM5 and Syn9 compared to phage T4. Orange genes are those found in phage T4. Blue shading is a hypervariable region localised between gp16 and gp17 in Syn9 (Millard *et al.*^2009^). The structural gene module that is common to all *T4likeviruses* is highlighted in green (Hambly *et al.*^2001^). (**B**) *T7likevirus* comparison of cyanophages Syn5 and P-SSP7, *Pseudoalteromonas* phage RIO-1 with phage T7. T7 genes found in other phages are teal coloured. The genomic islands identified in P-SSP7 are highlighted in yellow (Labrie *et al.*^2013^). (**C**) *N4likevirus* comparison of roseophage DSS3P2, *Pseudoalteromonas* phage pYD6-A and *Alteromonas* phage vB_AmaP_AD45-P1 with phage N4. Roseophage N4-like phages are represented by DSS3P2, which has genes that have been acquired by roseophages and localised at the same position (highlighted in red) (Chan *et al.*^2014^).

The sequencing of phage isolates has revealed common traits to phage genomes, including a modular genome structure, homology with phage infecting enteric bacteria and localisation of accessory genes within a specific region of the genome. The most common is a modular genome structure that is found in cyanophages, roseophages (phage infecting the roseobacter lineage) and pelagiphages infecting SAR11 and SAR116 (Millard *et al.*^2009^; Sullivan *et al.*^2010^; Kang *et al.*^2013^; Zhao *et al.*^2013^; Chan *et al.*^2014^). Another trait common to some groups of marine phages is a shared gene pool with phages infecting enteric pathogens (Fig. 3). This was first observed in the marine cyanophage S-PM2, a *T4likevirus* that infects *Synechococcus*, where a shared gene module that encodes for phage structural proteins was found in both S-PM2 and phage T4 infecting *Escherichia coli* (Hambly *et al.*^2001^) (Fig. 3).

Cyanophages have since become the most comprehensively studied group of marine phages, with those infecting cells of the genera *Synechococcus* and *Prochlorococcus* being predominantly studied (Hambly *et al.*^2001^; Chen and Lu 2002; Clokie *et al.*^2003^; Lindell *et al.*^2004^; Mann *et al.*^2005^; Sullivan *et al.*^2005^, ^2006^, ^2010^; Weigele *et al.*^2007^; Chenard and Suttle 2008; Millard *et al.*^2010^; Chan *et al.*^2011^; Labrie *et al.*^2013^). Currently, there are 193 genome sequences deposited in the ENA (Table S1, Supporting Information), the majority of which are part of the *T4likevirus* genus (Table S1, Supporting Information), sharing both morphological and genetic similarity (Hambly *et al.*^2001^; Weigele *et al.*^2007^; Millard *et al.*^2009^; Sullivan *et al.*^2010^), as well as an established set of core genes (Sullivan *et al.*^2010^; Ignacio-Espinoza and Sullivan 2012). Recently, an increased number of cyanophages of the podoviridae family has also been sequenced, revealing a conserved core of 12 genes and a close relationship to coliphage T7 (Labrie *et al.*^2013^). Whilst the genomes of podoviruses and myoviruses infecting cyanobacteria share very few genes, there are parallels with respect to the localisation of auxiliary metabolic genes (AMGs) in their genomes. Within the myoviridae, AMGs are found to be localised in hypervariable regions (Millard *et al.*^2004^) (Fig. 3). A similar pattern of AMG insertions within genomic islands was observed in cyanophages of the podoviridae family (Labrie *et al.*^2013^). An analogous system was also found for roseophages of the *N4likevirus* genus, whereby AMGs were found to be localised within specific regions (Chan *et al.*^2014^). Whilst AMGs are acquired by horizontal gene transfer, the mechanism that localises these genes to specific regions remains unknown.

In common with cyanophages, roseophages of the *N4likevirus* genus share a common gene pool with phages infecting enteric pathogens (Chan *et al.*^2014^). A comparison of 25 *N4likevirus* phages revealed 14 genes present in all roseophages and another nine genes present only in marine isolates, the latter likely including genes specifically required for infecting a marine host (Chan *et al.*^2014^).

Not all marine phages share genes with their non-marine counterparts. Unlike the phages discussed so far, phages infecting bacteria of the genus *Cellulophaga* share very few genes with any other phages (Holmfeldt *et al.*^2013^). The diversity of these 31 phages infecting *Cellulophaga baltica* (Table S1, Supporting Information) is far higher than observed for other marine phages and is comparable to the diversity observed in non-marine systems (Holmfeldt *et al.*^2013^). This single study exemplifies the benefit of sequencing cultured phage isolates to expand our understanding of phage genomes, given it identified 12 new genera and one new family from just 31 phage isolates (Holmfeldt *et al.*^2013^). The data garnered from the genomes of these *Cellulophaga* phages have subsequently been combined with experimental data, with a single nucleotide polymorphism in a gene encoding a putative tail spike protein linked to altered host range (Holmfeldt *et al.*^2014^).

## THE ‘SPECIES’ CONCEPT?

Sequencing the genome of phage isolates is also beginning to provide evidence for phage ‘species’. The idea of a phage ‘species’ concept derives from one of the first marine phages to be sequenced, SIO1, that infects *Roseobacter* SIO16, one of the limited number of marine phages to have been re-sequenced and re-annotated (Angly *et al.*^2009^). In a study conducted some 12 years after the original isolation of SIO1, a further four phages infecting *Roseobacter* SIO16 were isolated. In comparison to SIO1, these genomes were found to have 96%–98.4% average nucleotide identity (ANI), thus demonstrating that SIO1-like phages are stably maintained in the environment over ∼500 generations without any significant genomic re-arrangements (Angly *et al.*^2009^).

Whether a ‘species’ concept is valid for other phage-host systems remains to be determined. In cyanophages, no two completely sequenced genomes have similar ANI levels across their entire length, as observed for the SIO1-like phages (Millard *et al.*^2009^; Sullivan *et al.*^2010^). Yet, the sequencing of eight genes from 65 cyanophages isolated over a 10-year period confirmed the presence of phage ‘strains’ that are maintained within the environment (Marston and Amrich 2009). Furthermore, the use of viral tagging combined with metagenomics suggests that discrete populations of cyanophages are stable in the environment (Deng *et al.*^2014^).

## PHAGE AMGs

A common property of marine phage genomes is their propensity for carrying AMGs, which are thought to augment the metabolic potential of the host during the infection process (Breitbart *et al.*^2007^). This was first observed in roseophage SIO1, the first marine phage found to maintain a number of genes that were orthologues of host genes, including genes encoding a ribonucleotide reductase, a PhoH-like protein, thioredoxin and endodeoxyribonuclease I (Rohwer *et al.*^2000^).

As more marine phage genomes have been sequenced, it has become apparent that AMGs are a common feature. The most well-studied AMG is the *psbA* gene, encoding the D1 protein of Photosystem II (PSII), which was first reported in cyanophage S-PM2 (Mann *et al.*^2003^). It was subsequently found in the *Prochlorococcu*s-infecting phages P-SSM4 and P-SSM2 (Sullivan *et al.*^2005^) and is now known to be widespread in cyanophage isolates (Lindell *et al.*^2004^; Millard *et al.*^2004^, ^2009^; Sullivan *et al.*^2005^, ^2006^, ^2010^) and in the environment (Lindell *et al.*^2004^; Sharon *et al.*^2007^; Chenard and Suttle 2008; Zheng *et al.*^2013^). The genome of S-PM2 also revealed the presence of *psbD*, encoding the D2 protein of PSII (Mann *et al.*^2003^). By expression of *psbA* and *psbD*, cyanophages are thought to maintain the D1/D2 repair cycle, which are susceptible to photodamage if not replaced, and thus sustain photosynthetic function of their host during infection (Mann *et al.*^2003^). This is supported by the detection of both phage-derived *psbA* transcripts (Lindell *et al.*^2005^; Clokie *et al.*^2006^; Millard *et al.*^2010^) and D1 peptides from infected cells in laboratory studies (Lindell *et al.*^2005^). Moreover, in the environment *psbA* is a core gene in photic viromes and phage-derived transcripts are readily detected (Sharon *et al.*^2007^; Hurwitz, Brum and Sullivan 2014). Thus, the expression of phage-encoded genes was thought to directly contribute to CO_2_ fixation. However, there is evidence that the opposite occurs and CO_2_ is halted during early phage infection, whilst maintaining host photosynthetic electron transfer (Puxty *et al.*^2016^). *psbA* and *psbD* are not the only AMGs that can be broadly classified as influencing the photosynthetic function of their cyanobacterial hosts. A number of cyanophage genomes carry genes encoding proteins that may inhibit the Calvin cycle, encode a variety of electron transport components or are involved in pigment biosynthesis and central carbon metabolism (Millard *et al.*^2004^, ^2009^; Sullivan *et al.*^2005^, ^2010^; Weigele *et al.*^2007^; Sabehi *et al.*^2012^; Dekel-Bird *et al.*^2013^; Frank *et al.*^2013^). In addition, metagenomics has revealed a cassette of genes encoding a putative functional Photosystem I (PSI) (Sharon *et al.*^2009^), with some phages possessing genes encoding both photosystems (Alperovitch-Lavy *et al.*^2011^). However, no cultured phage isolates have, as yet, been found to contain a complete PSI and PSII cassette.

The roles of many of these photosynthesis-related AMGs have been comprehensively reviewed elsewhere (see Puxty *et al.*^2015^). Whilst there is limited functional data for most of these photosynthesis-related genes, it is thought that these AMGs generally provide the same function as their cyanobacterial orthologues, as is the case with the photosynthesis-related genes *ho1*, *petF* and *pcyA* (Dammeyer *et al.*^2008^)*.* In the case of the phage-encoded PebS additional function is provided, as PebS catalyses a reaction that normally requires two host proteins (PebA and PebB) (Dammeyer *et al.*^2008^). The carriage of *pcyA* and *pebS* is thought to provide a fitness advantage to the phage, through an as yet undescribed mechanism, since these genes were found in phage-infecting *Prochlorococcus* which do not possess phycobilisome complexes. The puzzling role of genes related to phycobilin biosynthesis in phages is further confounded by the recent discovery and biochemical characterisation of a PcyA homologue within a phage that likely infects an alphaproteobacteria, thus suggesting a role for phycobilins outside cyanobacteria (Ledermann, Beja and Frankenberg-Dinkel 2016).

Beyond photosynthesis and carbon metabolism, AMGs that may alter other biogeochemical cycles have also been discovered. Three genes with a possible role in P metabolism have been identified so far: *phoH*, which is widespread in marine phages (Goldsmith *et al.*^2011^) and a core-gene in T4-like phages (Ignacio-Espinoza and Sullivan 2012), is known to be part of the phosphate regulon in *Escherichia coli* (Kim *et al*. 1993), but its function in phages is unclear (Goldsmith *et al.*^2011^). Other genes related to P metabolism within cyanophages have also been identified: *pstS* encoding a potential periplasmic phosphate binding protein and *phoA* encoding a putative alkaline phosphatase (Sullivan *et al.*^2010^). Intriguingly, only *pstS* is overexpressed during infection of a P-deplete host (Lin, Ding and Zeng 2016).

Recent research suggests that phages may also influence the sulphur cycle. The SAR116 infecting phage HMO-2011 contains a gene encoding a putative hydroxylase α-subunit of methanesulfonate monooxygenase (MsmA) (Kang *et al.*^2013^). Methanesulfonic acid (MSA) is an important intermediate in the sulphur cycle (Kelly and Murrell 1999). As MSA can be oxidised to sulphite and formaldehyde (Kelly and Murrell 1999) and SAR116 contains the required genes to uptake and oxidise MSA and further metabolise formaldehyde (Kang *et al.*^2013^), the phage-encoded MsmA is speculated to play a role in the initial oxidation of MSA. Genes encoding the α and γ-subunits of the reverse dissimilatory sulphite reductase (*rdsR*) are found to be widespread in phages infecting SUP05, and are thought to provide roles in the oxidation of sulphur to sulphite, thus providing phage with an energy source for the infection process (Anantharaman *et al.*^2014^).

Whilst the evidence for phage encoded genes influencing global biogeochemical cycles is increasing, there are fewer insights into the role of phage AMGs altering the virulence of their hosts. The best example is the filamentous phage CTX, which carries the toxin encoding genes responsible for the full virulence of *Vibrio cholerae* (Waldor and Mekalanos 1996). Whilst *V. cholerae* is found in freshwater and brackish environments, it is also globally distributed in the marine environment (Escobar *et al.*^2015^). In addition, other vibriophages have been isolated that can alter the virulence of their host. Three phages infecting *V. harveyi* have been sequenced to date*—*two siphoviruses (VHS1 & SIO-2) (Khemayan *et al.*^2012^) and a myovirus (VHML) (Oakey, Cullen and Owens 2002). Despite having very different genome content, both VHML and VHS1 are thought to have the potential to enhance the virulence of their host by the carriage of toxin (Khemayan *et al.*^2012^) or toxin-associated genes (Oakey, Cullen and Owens 2002).

Viral metagenomic surveys are currently identifying an increasing diversity of AMGs. The challenge then is to link these AMGs with specific phage genomes and derive phage-host systems. This will enable experimental functional analysis of these genes that likely play key roles altering host metabolism/virulence during infection and with potentially important knock-on effects for the functioning of global biogeochemical cycles.

## CONCLUSIONS

In the last decade, whilst there have been rapid advances in sequencing technologies, these advances have not been fully exploited in the field of marine phage genomics, with both the total number and diversity of phage genomes lagging in comparison to their bacterial hosts. To fully interpret viral metagenomes and more completely understand the role of phage in the marine environment, a greater emphasis needs to be placed on isolating phage to a range of marine bacterial genera and sequencing their genomes, ultimately providing new model systems for experimental testing of phage–host interactions. The latter is readily achievable and affordable with current sequencing platforms. Even with the relatively small number of marine phage genomes completed, rapid progress has been made; hence, the future can only surely bring further fascinating insights into the role phages play in marine systems.

## Supplementary Material

Supplementary Data
