# Supplementary material for: Marine phage genomics: the tip of the iceberg
Source: FEMS Microbiol Lett. 2016 Jun 22;363(15):fnw158. doi: 10.1093/femsle/fnw158 (PMC4928673; doi:10.1093/femsle/fnw158)
Supplement: Supplementary Data [file fnw158_supplementary_data.zip › Table_S1.docx]

Table S1 Marine phages identified within the ENA phage database. The genus of the host bacterium, number of phage that infect the host, classification of the phage at the level of: order, family, subfamily and genus are detailed long with the phage name

| **Host bacterium (Genera)** | **Number of phage genomes** | **Accession Number(s)** | **Order** | **Family** | **Subfamily** | **Genus** | **Phage name** |
| --- | --- | --- | --- | --- | --- | --- | --- |
| *Alteromonas* | 3 | KF005318.1 | Caudovirales | Podoviridae |  |  | vB_AmaP_AD45-P |
| *Alteromonas* | 3 | KF005319.1 | Caudovirales | Podoviridae |  |  | vB_AmaP_AD45-P |
| *Alteromonas* | 3 | KF005320.1 | Caudovirales | Podoviridae |  |  | vB_AmaP_AD45-P |
| *Candidatus Pelagibacter* | 4 | KC465899.1 | Caudovirales | Myoviridae |  |  | HTVC008M |
| *Candidatus Pelagibacter* | 4 | KC465898.1 | Caudovirales | Podoviridae |  |  | HTVC010P |
| *Candidatus Pelagibacter* | 4 | KC465900.1 | Caudovirales | Podoviridae |  |  | HTVC019P |
| *Candidatus Pelagibacter* | 4 | KC465901.1 | Caudovirales | Podoviridae |  |  | HTVC019P |
| *Cellulophaga* | 31 | KC821604.1 | Caudovirales | Siphoviridae |  |  | phiST |
| *Cellulophaga* | 31 | KC821605.1 |  |  |  |  | phi48:2 |
| *Cellulophaga* | 31 | KC821606.1 |  | Microviridae |  |  | phi12:2 |
| *Cellulophaga* | 31 | KC821607.1 | Caudovirales | Siphoviridae |  |  | phi19:1 |
| *Cellulophaga* | 31 | KC821608.1 | Caudovirales | Podoviridae |  |  | phi19:3 |
| *Cellulophaga* | 31 | KC821609.1 | Caudovirales | Podoviridae |  |  | phi17:2 |
| *Cellulophaga* | 31 | KC821610.1 | Caudovirales | Myoviridae |  |  | phiSM |
| *Cellulophaga* | 31 | KC821611.1 | Caudovirales | Siphoviridae |  |  | phi46:1 |
| *Cellulophaga* | 31 | KC821612.1 | Caudovirales | Podoviridae |  |  | phi38:1 |
| *Cellulophaga* | 31 | KC821613.1 | Caudovirales | Siphoviridae |  |  | phi12:1 |
| *Cellulophaga* | 31 | KC821614.1 | Caudovirales | Podoviridae |  |  | phi38:1 |
| *Cellulophaga* | 31 | KC821615.1 | Caudovirales | Siphoviridae |  |  | phi12:1 |
| *Cellulophaga* | 31 | KC821616.1 | Caudovirales | Myoviridae |  |  | phiSM |
| *Cellulophaga* | 31 | KC821617.1 | Caudovirales | Siphoviridae |  |  | phi17:1 |
| *Cellulophaga* | 31 | KC821618.1 | Caudovirales | Siphoviridae |  |  | phi10:1 |
| *Cellulophaga* | 31 | KC821619.1 | Caudovirales | Siphoviridae |  |  | phi18:1 |
| *Cellulophaga* | 31 | KC821620.1 | Caudovirales | Podoviridae |  |  | phi18:3 |
| *Cellulophaga* | 31 | KC821621.1 | Caudovirales | Siphoviridae |  |  | phiST |
| *Cellulophaga* | 31 | KC821622.1 | Caudovirales | Podoviridae |  |  | phi46:3 |
| *Cellulophaga* | 31 | KC821623.1 |  | Microviridae |  |  | phi12a:1 |
| *Cellulophaga* | 31 | KC821624.1 | Caudovirales | Podoviridae |  |  | phi14:2 |
| *Cellulophaga* | 31 | KC821625.1 | Caudovirales | Siphoviridae |  |  | phiST |
| *Cellulophaga* | 31 | KC821626.1 | Caudovirales | Siphoviridae |  |  | phi39:1 |
| *Cellulophaga* | 31 | KC821627.1 | Caudovirales | Siphoviridae |  |  | phi18:1 |
| *Cellulophaga* | 31 | KC821628.1 |  | Microviridae |  |  | phi12a:1 |
| *Cellulophaga* | 31 | KC821629.1 | Caudovirales | Myoviridae |  |  | phiSM |
| *Cellulophaga* | 31 | KC821630.1 | Caudovirales | Myoviridae |  |  | phiSM |
| *Cellulophaga* | 31 | KC821631.1 |  | Microviridae |  |  | phi12a:1 |
| *Cellulophaga* | 31 | KC821632.1 | Caudovirales | Podoviridae |  |  | phi4:1 |
| *Cellulophaga* | 31 | KC821633.1 | Caudovirales | Podoviridae |  |  | phi13:2 |
| *Cellulophaga* | 31 | KC821634.1 | Caudovirales | Myoviridae |  |  | phiSM |
| *Dinoroseobacter* | 2 | KJ621082.2 | Caudovirales | Podoviridae |  | *N4likevirus* | DFL12phi1 |
| *Dinoroseobacter* | 2 | KJ803031.1 | Caudovirales | Podoviridae |  | *N4likevirus* | vBDshPR2C |
| *Flavobacterium* | 2 | KM873719.2 | Caudovirales | Myoviridae |  |  | FCL-2 |
| *Flavobacterium* | 2 | KJ018210.1 | Caudovirales | Myoviridae |  |  | 1 32 |
| *Geobacillus* | 1 | GU568037.1 |  |  |  |  | D6E |
| *Halomonas* | 1 | EU399241.1 | Caudovirales | Myoviridae |  | *Hapunalikevirus* | phiHAP-1 |
| Host not cultured - DCM of the Mediterranean Sea | 207 | AP013358.1-AP013565.1 |  |  |  |  |  |
| *Idiomarina* | 2 | KJ847052.1 |  |  |  |  | 1N2-2 |
| *Idiomarina* | 2 | KM226324.1 | Caudovirales | Siphoviridae |  |  | phi1M2 2 |
| *Persicivirga* | 2 | JQ823122.1 |  |  |  |  | P12024S |
| *Persicivirga* | 2 | JQ823123.1 |  |  |  |  | P12024L |
| *Prochlorococcus* | 19 | KP211958.1 | Caudovirales | Myoviridae |  |  | P TIM40 |
| *Prochlorococcus* | 19 | AY939843.2 | Caudovirales | Podoviridae | Autographivirinae |  | P-SSP7 |
| *Prochlorococcus* | 19 | AY939844.2 | Caudovirales | Myoviridae | Tevenvirinae | *T4likevirus* | P-SSM2 |
| *Prochlorococcus* | 19 | AY940168.2 | Caudovirales | Myoviridae | Tevenvirinae | *T4likevirus* | P-SSM4 |
| *Prochlorococcus* | 19 | GQ334450.1 | Caudovirales | Siphoviridae |  |  | PSS2 |
| *Prochlorococcus* | 19 | GU071092.1 | Caudovirales | Myoviridae | Tevenvirinae | *T4likevirus* | P-SSM2 |
| *Prochlorococcus* | 19 | GU071099.1 | Caudovirales | Myoviridae |  |  | P-RSM4 |
| *Prochlorococcus* | 19 | GU071100.1 | Caudovirales | Podoviridae | Autographivirinae |  | 9515-10a |
| *Prochlorococcus* | 19 | GU071101.1 | Caudovirales | Myoviridae |  |  | P-HM1 |
| *Prochlorococcus* | 19 | GU071102.1 | Caudovirales | Podoviridae | Autographivirinae |  | NATL1A-7 |
| *Prochlorococcus* | 19 | GU071103.1 | Caudovirales | Myoviridae |  |  | P-SSM7 |
| *Prochlorococcus* | 19 | GU071104.1 | Caudovirales | Podoviridae | Autographivirinae |  | NATL2A-133 |
| *Prochlorococcus* | 19 | GU071105.1 | Caudovirales |  |  |  | Syn1 |
| *Prochlorococcus* | 19 | GU071107.1 | Caudovirales |  |  |  | P-SSP2 |
| *Prochlorococcus* | 19 | GU071108.1 | Caudovirales |  |  |  | Syn33 |
| *Prochlorococcus* | 19 | GU075905.1 | Caudovirales | Myoviridae |  |  | P-HM2 |
| *Prochlorococcus* | 19 | HQ634152.1 | Caudovirales | Podoviridae | Autographivirinae |  | 9515-10a |
| *Prochlorococcus* | 19 | HQ634174.1 | Caudovirales | Myoviridae |  |  | MED4-213 |
| *Prochlorococcus* | 19 | KF598865.1 | Caudovirales | Myoviridae |  |  | P-HM1 |
| *Pseudoalteromonas* | 4 | AF155037.1 |  | Corticoviridae | | *Corticovirus* | PM2 |
| *Pseudoalteromonas* | 4 | HM588722.1 | Caudovirales | Siphoviridae |  |  | H105/1 |
| *Pseudoalteromonas* | 4 | KC542353.1 | Caudovirales | Siphoviridae |  |  | TW1 |
| *Pseudoalteromonas* | 4 | KC751414.1 | Caudovirales | Podoviridae |  |  | RIO-1 |
| *Puniceispirillum* | 1 | GU557055.1 | Caudovirales | Podoviridae |  |  | HMO-2011 |
| *Roseobacter* | 2 | HM151342.1 | Caudovirales | Siphoviridae |  |  | RDJL Phi 1 |
| *Roseobacter* | 2 | AF189021.1 | Caudovirales | Podoviridae |  |  | SIO1 |
| *Ruegeria* | 1 | KM581061.1 | Caudovirales | Siphoviridae |  |  | DSS3 P1 |
| *Shewanella* | 5 | KJ018209.1 | Caudovirales | Myoviridae |  |  | 1-4 |
| *Shewanella* | 5 | KJ018211.1 | Caudovirales | Myoviridae |  |  | 1-40 |
| *Shewanella* | 5 | AHK11647.1 | Caudovirales | Myoviridae |  |  | 1-41 |
| *Shewanella* | 5 | KJ018213.1 | Caudovirales | Myoviridae |  |  | 1-44 |
| *Shewanella* | 5 | KJ018214.1 | Caudovirales | Myoviridae |  |  | 3-49 |
| *Silicibacter* | 1 | FJ591093.1 | Caudovirales | Podoviridae |  | *N4likevirus* | DSS3phi2 |
| *Alpha-proteobacterium* | 1 | AY576273.1 | Caudovirales | Siphoviridae |  | *Yualikevirus* | phiJL001 |
| *Sulfitobacter* | 1 | FJ591094.1 | Caudovirales | Podoviridae |  | *N4likevirus* | EE36phi1 |
| *Sulfitobacter* | 1 | KM233261.1 |  |  |  |  | NYA-2014a |
| *Synechococcus* | 147 | AJ630128.1 | Caudovirales | Myoviridae | Tevenvirinae | *T4likevirus* | S-PM2 |
| *Synechococcus* | 147 | DQ149023.2 | Caudovirales | Myoviridae | Tevenvirinae | *T4likevirus* | Syn9 |
| *Synechococcus* | 147 | EF372997.1 | Caudovirales | Podoviridae | Autographivirinae |  | Syn5 |
| *Synechococcus* | 147 | FM207411.1 | Caudovirales | Myoviridae | Tevenvirinae | *T4likevirus* | S-RSM4 |
| *Synechococcus* | 147 | GU071094.1 | Caudovirales | Myoviridae |  |  | S-SM1 |
| *Synechococcus* | 147 | GU071095.1 | Caudovirales | Myoviridae |  |  | S-SM2 |
| *Synechococcus* | 147 | GU071096.1 | Caudovirales | Myoviridae |  |  | S-ShM2 |
| *Synechococcus* | 147 | GU071097.1 | Caudovirales | Myoviridae |  |  | S-SSM5 |
| *Synechococcus* | 147 | GU071098.1 | Caudovirales | Myoviridae |  |  | S-SSM7 |
| *Synechococcus* | 147 | GU936714.1 | Caudovirales | Siphoviridae |  |  | S-CBS2 |
| *Synechococcus* | 147 | GU936715.1 | Caudovirales | Siphoviridae |  |  | S-CBS3 |
| *Synechococcus* | 147 | HM480106.1 | Caudovirales | Siphoviridae |  |  | S-CBS1 |
| *Synechococcus* | 147 | HQ317290.1 | Caudovirales | Myoviridae |  |  | S-RIM2 |
| *Synechococcus* | 147 | HQ317291.1 | Caudovirales | Myoviridae |  |  | S-RIM2 |
| *Synechococcus* | 147 | HQ317292.1 | Caudovirales | Myoviridae |  |  | S-RIM2 |
| *Synechococcus* | 147 | HQ317385.1 | Caudovirales | Myoviridae |  |  | S-RIM8 |
| *Synechococcus* | 147 | HQ615693.1 | Caudovirales | Myoviridae |  |  | S-CRM01 |
| *Synechococcus* | 147 | HQ634178.1 | Caudovirales | Myoviridae |  |  | S-CAM8 |
| *Synechococcus* | 147 | HQ698895.1 | Caudovirales | Siphoviridae |  |  | S-CBS4 |
| *Synechococcus* | 147 | JF974288.1 | Caudovirales | Myoviridae |  |  | S-RIM8 |
| *Synechococcus* | 147 | JF974289.1 | Caudovirales | Myoviridae |  |  | S-RIM8 |
| *Synechococcus* | 147 | JN371768.1 | Caudovirales | Myoviridae | Tevenvirinae | *T4likevirus* | ACG-2014c |
| *Synechococcus* | 147 | JN371769.1 | Caudovirales | Myoviridae | Tevenvirinae | *T4likevirus* | metaG-MbCM1 |
| *Synechococcus* | 147 | KF156338.1 | Caudovirales | Myoviridae | Tevenvirinae | *T4likevirus* | ACG-2014h |
| *Synechococcus* | 147 | KF156339.1 | Caudovirales | Myoviridae | Tevenvirinae | *T4likevirus* | ACG-2014c |
| *Synechococcus* | 147 | KF156340.1 | Caudovirales | Myoviridae | Tevenvirinae | *T4likevirus* | S-MbCM100 |
| *Synechococcus* | 147 | KJ019135 | Caudovirales | Myoviridae | Tevenvirinae | *T4likevirus* | ACG-2014a isolate Syn7803C101 |
| *Synechococcus* | 147 | KJ019137 | Caudovirales | Myoviridae | Tevenvirinae | *T4likevirus* | ACG-2014a isolate Syn7803C104 |
| *Synechococcus* | 147 | KJ019138 | Caudovirales | Myoviridae | Tevenvirinae | *T4likevirus* | ACG-2014a isolate Syn7803C107 |
| *Synechococcus* | 147 | KJ019153 | Caudovirales | Myoviridae | Tevenvirinae | *T4likevirus* | ACG-2014a isolate Syn7803C26 |
| *Synechococcus* | 147 | KJ019157 | Caudovirales | Myoviridae | Tevenvirinae | *T4likevirus* | ACG-2014a isolate Syn7803C31 |
| *Synechococcus* | 147 | KJ019158 | Caudovirales | Myoviridae | Tevenvirinae | *T4likevirus* | ACG-2014a isolate Syn7803C33 |
| *Synechococcus* | 147 | KJ019163 | Caudovirales | Myoviridae | Tevenvirinae | *T4likevirus* | ACG-2014a isolate Syn7803C38 |
| *Synechococcus* | 147 | KJ019026 | Caudovirales | Myoviridae | Tevenvirinae | *T4likevirus* | ACG-2014a isolate Syn7803C42 |
| *Synechococcus* | 147 | KJ019030 | Caudovirales | Myoviridae | Tevenvirinae | *T4likevirus* | ACG-2014a isolate Syn7803C47 |
| *Synechococcus* | 147 | KJ019033 | Caudovirales | Myoviridae | Tevenvirinae | *T4likevirus* | ACG-2014a isolate Syn7803C53 |
| *Synechococcus* | 147 | KJ019038 | Caudovirales | Myoviridae | Tevenvirinae | *T4likevirus* | ACG-2014a isolate Syn7803C59 |
| *Synechococcus* | 147 | KJ019039 | Caudovirales | Myoviridae | Tevenvirinae | *T4likevirus* | ACG-2014a isolate Syn7803C60 |
| *Synechococcus* | 147 | KJ019055 | Caudovirales | Myoviridae | Tevenvirinae | *T4likevirus* | ACG-2014a isolate Syn7803C86 |
| *Synechococcus* | 147 | KJ019065 | Caudovirales | Myoviridae | Tevenvirinae | *T4likevirus* | ACG-2014a isolate Syn7803C99 |
| *Synechococcus* | 147 | KJ019088 | Caudovirales | Myoviridae | Tevenvirinae | *T4likevirus* | ACG-2014a isolate Syn7803US1 |
| *Synechococcus* | 147 | KJ019067 | Caudovirales | Myoviridae | Tevenvirinae | *T4likevirus* | ACG-2014a isolate Syn7803US101 |
| *Synechococcus* | 147 | KJ019068 | Caudovirales | Myoviridae | Tevenvirinae | *T4likevirus* | ACG-2014a isolate Syn7803US102 |
| *Synechococcus* | 147 | KJ019076 | Caudovirales | Myoviridae | Tevenvirinae | *T4likevirus* | ACG-2014a isolate Syn7803US112 |
| *Synechococcus* | 147 | KJ019081 | Caudovirales | Myoviridae | Tevenvirinae | *T4likevirus* | ACG-2014a isolate Syn7803US117 |
| *Synechococcus* | 147 | KJ019084 | Caudovirales | Myoviridae | Tevenvirinae | *T4likevirus* | ACG-2014a isolate Syn7803US123 |
| *Synechococcus* | 147 | KJ019087 | Caudovirales | Myoviridae | Tevenvirinae | *T4likevirus* | ACG-2014a isolate Syn7803US19 |
| *Synechococcus* | 147 | KJ019114 | Caudovirales | Myoviridae | Tevenvirinae | *T4likevirus* | ACG-2014a isolate Syn7803US60 |
| *Synechococcus* | 147 | KJ019116 | Caudovirales | Myoviridae | Tevenvirinae | *T4likevirus* | ACG-2014a isolate Syn7803US62 |
| *Synechococcus* | 147 | KJ019122 | Caudovirales | Myoviridae | Tevenvirinae | *T4likevirus* | ACG-2014a isolate Syn7803US79 |
| *Synechococcus* | 147 | KJ019134 | Caudovirales | Myoviridae | Tevenvirinae | *T4likevirus* | ACG-2014b isolate Syn7803C100 |
| *Synechococcus* | 147 | KJ019154 | Caudovirales | Myoviridae | Tevenvirinae | *T4likevirus* | ACG-2014b isolate Syn7803C28 |
| *Synechococcus* | 147 | KJ019161 | Caudovirales | Myoviridae | Tevenvirinae | *T4likevirus* | ACG-2014b isolate Syn7803C36 |
| *Synechococcus* | 147 | KJ019040 | Caudovirales | Myoviridae | Tevenvirinae | *T4likevirus* | ACG-2014b isolate Syn7803C61 |
| *Synechococcus* | 147 | KJ019041 | Caudovirales | Myoviridae | Tevenvirinae | *T4likevirus* | ACG-2014b isolate Syn7803C66 |
| *Synechococcus* | 147 | KJ019042 | Caudovirales | Myoviridae | Tevenvirinae | *T4likevirus* | ACG-2014b isolate Syn7803C67 |
| *Synechococcus* | 147 | KJ019043 | Caudovirales | Myoviridae | Tevenvirinae | *T4likevirus* | ACG-2014b isolate Syn7803C68 |
| *Synechococcus* | 147 | KJ019044 | Caudovirales | Myoviridae | Tevenvirinae | *T4likevirus* | ACG-2014b isolate Syn7803C69 |
| *Synechococcus* | 147 | KJ019049 | Caudovirales | Myoviridae | Tevenvirinae | *T4likevirus* | ACG-2014b isolate Syn7803C76 |
| *Synechococcus* | 147 | KJ019051 | Caudovirales | Myoviridae | Tevenvirinae | *T4likevirus* | ACG-2014b isolate Syn7803C78 |
| *Synechococcus* | 147 | KJ019060 | Caudovirales | Myoviridae | Tevenvirinae | *T4likevirus* | ACG-2014b isolate Syn7803C91 |
| *Synechococcus* | 147 | KJ019061 | Caudovirales | Myoviridae | Tevenvirinae | *T4likevirus* | ACG-2014b isolate Syn7803C92 |
| *Synechococcus* | 147 | KJ019104 | Caudovirales | Myoviridae | Tevenvirinae | *T4likevirus* | ACG-2014b isolate Syn7803US49 |
| *Synechococcus* | 147 | KJ019108 | Caudovirales | Myoviridae | Tevenvirinae | *T4likevirus* | ACG-2014b isolate Syn7803US53 |
| *Synechococcus* | 147 | KJ019109 | Caudovirales | Myoviridae | Tevenvirinae | *T4likevirus* | ACG-2014b isolate Syn7803US54 |
| *Synechococcus* | 147 | KJ019110 | Caudovirales | Myoviridae | Tevenvirinae | *T4likevirus* | ACG-2014b isolate Syn7803US56 |
| *Synechococcus* | 147 | KJ019132 | Caudovirales | Myoviridae | Tevenvirinae | *T4likevirus* | ACG-2014b isolate Syn9311C1 |
| *Synechococcus* | 147 | KJ019133 | Caudovirales | Myoviridae | Tevenvirinae | *T4likevirus* | ACG-2014b isolate Syn9311C4 |
| *Synechococcus* | 147 | JN371768 | Caudovirales | Myoviridae | Tevenvirinae | *T4likevirus* | ACG-2014c |
| *Synechococcus* | 147 | KJ019027 | Caudovirales | Myoviridae | Tevenvirinae | *T4likevirus* | ACG-2014c isolate Syn7803C43 |
| *Synechococcus* | 147 | KJ019063 | Caudovirales | Myoviridae | Tevenvirinae | *T4likevirus* | ACG-2014c isolate Syn7803C97 |
| *Synechococcus* | 147 | KJ019064 | Caudovirales | Myoviridae | Tevenvirinae | *T4likevirus* | ACG-2014c isolate Syn7803C98 |
| *Synechococcus* | 147 | KJ019128 | Caudovirales | Myoviridae | Tevenvirinae | *T4likevirus* | ACG-2014c isolate Syn7803US88 |
| *Synechococcus* | 147 | KJ019136 | Caudovirales | Myoviridae | Tevenvirinae | *T4likevirus* | ACG-2014d isolate Syn7803C102 |
| *Synechococcus* | 147 | KJ019139 | Caudovirales | Myoviridae | Tevenvirinae | *T4likevirus* | ACG-2014d isolate Syn7803C108 |
| *Synechococcus* | 147 | KJ019140 | Caudovirales | Myoviridae | Tevenvirinae | *T4likevirus* | ACG-2014d isolate Syn7803C109 |
| *Synechococcus* | 147 | KJ019160 | Caudovirales | Myoviridae | Tevenvirinae | *T4likevirus* | ACG-2014d isolate Syn7803C35 |
| *Synechococcus* | 147 | KJ019162 | Caudovirales | Myoviridae | Tevenvirinae | *T4likevirus* | ACG-2014d isolate Syn7803C37 |
| *Synechococcus* | 147 | KJ019164 | Caudovirales | Myoviridae | Tevenvirinae | *T4likevirus* | ACG-2014d isolate Syn7803C39 |
| *Synechococcus* | 147 | KJ019165 | Caudovirales | Myoviridae | Tevenvirinae | *T4likevirus* | ACG-2014d isolate Syn7803C40 |
| *Synechococcus* | 147 | KJ019028 | Caudovirales | Myoviridae | Tevenvirinae | *T4likevirus* | ACG-2014d isolate Syn7803C45 |
| *Synechococcus* | 147 | KJ019029 | Caudovirales | Myoviridae | Tevenvirinae | *T4likevirus* | ACG-2014d isolate Syn7803C46 |
| *Synechococcus* | 147 | KJ019031 | Caudovirales | Myoviridae | Tevenvirinae | *T4likevirus* | ACG-2014d isolate Syn7803C48 |
| *Synechococcus* | 147 | KJ019032 | Caudovirales | Myoviridae | Tevenvirinae | *T4likevirus* | ACG-2014d isolate Syn7803C49 |
| *Synechococcus* | 147 | KJ019034 | Caudovirales | Myoviridae | Tevenvirinae | *T4likevirus* | ACG-2014d isolate Syn7803C54 |
| *Synechococcus* | 147 | KJ019035 | Caudovirales | Myoviridae | Tevenvirinae | *T4likevirus* | ACG-2014d isolate Syn7803C55 |
| *Synechococcus* | 147 | KJ019036 | Caudovirales | Myoviridae | Tevenvirinae | *T4likevirus* | ACG-2014d isolate Syn7803C57 |
| *Synechococcus* | 147 | KJ019046 | Caudovirales | Myoviridae | Tevenvirinae | *T4likevirus* | ACG-2014d isolate Syn7803C72 |
| *Synechococcus* | 147 | KJ019047 | Caudovirales | Myoviridae | Tevenvirinae | *T4likevirus* | ACG-2014d isolate Syn7803C73 |
| *Synechococcus* | 147 | KJ019048 | Caudovirales | Myoviridae | Tevenvirinae | *T4likevirus* | ACG-2014d isolate Syn7803C75 |
| *Synechococcus* | 147 | KJ019050 | Caudovirales | Myoviridae | Tevenvirinae | *T4likevirus* | ACG-2014d isolate Syn7803C77 |
| *Synechococcus* | 147 | KJ019057 | Caudovirales | Myoviridae | Tevenvirinae | *T4likevirus* | ACG-2014d isolate Syn7803C89 |
| *Synechococcus* | 147 | KJ019062 | Caudovirales | Myoviridae | Tevenvirinae | *T4likevirus* | ACG-2014d isolate Syn7803C93 |
| *Synechococcus* | 147 | KJ019070 | Caudovirales | Myoviridae | Tevenvirinae | *T4likevirus* | ACG-2014d isolate Syn7803US104 |
| *Synechococcus* | 147 | KJ019072 | Caudovirales | Myoviridae | Tevenvirinae | *T4likevirus* | ACG-2014d isolate Syn7803US108 |
| *Synechococcus* | 147 | KJ019073 | Caudovirales | Myoviridae | Tevenvirinae | *T4likevirus* | ACG-2014d isolate Syn7803US109 |
| *Synechococcus* | 147 | KJ019074 | Caudovirales | Myoviridae | Tevenvirinae | *T4likevirus* | ACG-2014d isolate Syn7803US110 |
| *Synechococcus* | 147 | KJ019075 | Caudovirales | Myoviridae | Tevenvirinae | *T4likevirus* | ACG-2014d isolate Syn7803US111 |
| *Synechococcus* | 147 | KJ019077 | Caudovirales | Myoviridae | Tevenvirinae | *T4likevirus* | ACG-2014d isolate Syn7803US113 |
| *Synechococcus* | 147 | KJ019078 | Caudovirales | Myoviridae | Tevenvirinae | *T4likevirus* | ACG-2014d isolate Syn7803US114 |
| *Synechococcus* | 147 | KJ019079 | Caudovirales | Myoviridae | Tevenvirinae | *T4likevirus* | ACG-2014d isolate Syn7803US115 |
| *Synechococcus* | 147 | KJ019080 | Caudovirales | Myoviridae | Tevenvirinae | *T4likevirus* | ACG-2014d isolate Syn7803US116 |
| *Synechococcus* | 147 | KJ019083 | Caudovirales | Myoviridae | Tevenvirinae | *T4likevirus* | ACG-2014d isolate Syn7803US122 |
| *Synechococcus* | 147 | KJ019113 | Caudovirales | Myoviridae | Tevenvirinae | *T4likevirus* | ACG-2014d isolate Syn7803US5 |
| *Synechococcus* | 147 | KJ019112 | Caudovirales | Myoviridae | Tevenvirinae | *T4likevirus* | ACG-2014d isolate Syn7803US59 |
| *Synechococcus* | 147 | KJ019115 | Caudovirales | Myoviridae | Tevenvirinae | *T4likevirus* | ACG-2014d isolate Syn7803US61 |
| *Synechococcus* | 147 | KJ019117 | Caudovirales | Myoviridae | Tevenvirinae | *T4likevirus* | ACG-2014d isolate Syn7803US63 |
| *Synechococcus* | 147 | KJ019118 | Caudovirales | Myoviridae | Tevenvirinae | *T4likevirus* | ACG-2014d isolate Syn7803US64 |
| *Synechococcus* | 147 | KJ019119 | Caudovirales | Myoviridae | Tevenvirinae | *T4likevirus* | ACG-2014d isolate Syn7803US65 |
| *Synechococcus* | 147 | KJ019120 | Caudovirales | Myoviridae | Tevenvirinae | *T4likevirus* | ACG-2014d isolate Syn7803US71 |
| *Synechococcus* | 147 | KJ019121 | Caudovirales | Myoviridae | Tevenvirinae | *T4likevirus* | ACG-2014d isolate Syn7803US78 |
| *Synechococcus* | 147 | KJ019124 | Caudovirales | Myoviridae | Tevenvirinae | *T4likevirus* | ACG-2014d isolate Syn7803US80 |
| *Synechococcus* | 147 | KJ019125 | Caudovirales | Myoviridae | Tevenvirinae | *T4likevirus* | ACG-2014d isolate Syn7803US82 |
| *Synechococcus* | 147 | KJ019126 | Caudovirales | Myoviridae | Tevenvirinae | *T4likevirus* | ACG-2014d isolate Syn7803US83 |
| *Synechococcus* | 147 | KJ019127 | Caudovirales | Myoviridae | Tevenvirinae | *T4likevirus* | ACG-2014d isolate Syn7803US85 |
| *Synechococcus* | 147 | KJ019129 | Caudovirales | Myoviridae | Tevenvirinae | *T4likevirus* | ACG-2014d isolate Syn7803US89 |
| *Synechococcus* | 147 | KJ019130 | Caudovirales | Myoviridae | Tevenvirinae | *T4likevirus* | ACG-2014d isolate Syn7803US94 |
| *Synechococcus* | 147 | KJ019131 | Caudovirales | Myoviridae | Tevenvirinae | *T4likevirus* | ACG-2014d isolate Syn7803US95 |
| *Synechococcus* | 147 | KJ019156 | Caudovirales | Myoviridae | Tevenvirinae | *T4likevirus* | ACG-2014e isolate Syn7803C2 |
| *Synechococcus* | 147 | KJ019054 | Caudovirales | Myoviridae | Tevenvirinae | *T4likevirus* | ACG-2014e isolate Syn7803C85 |
| *Synechococcus* | 147 | KJ019094 | Caudovirales | Myoviridae | Tevenvirinae | *T4likevirus* | ACG-2014e isolate Syn7803US33 |
| *Synechococcus* | 147 | KJ019141 | Caudovirales | Myoviridae | Tevenvirinae | *T4likevirus* | ACG-2014f isolate Syn7803C10 |
| *Synechococcus* | 147 | KJ019142 | Caudovirales | Myoviridae | Tevenvirinae | *T4likevirus* | ACG-2014f isolate Syn7803C11 |
| *Synechococcus* | 147 | KJ019143 | Caudovirales | Myoviridae | Tevenvirinae | *T4likevirus* | ACG-2014f isolate Syn7803C12 |
| *Synechococcus* | 147 | KJ019144 | Caudovirales | Myoviridae | Tevenvirinae | *T4likevirus* | ACG-2014f isolate Syn7803C14 |
| *Synechococcus* | 147 | KJ019145 | Caudovirales | Myoviridae | Tevenvirinae | *T4likevirus* | ACG-2014f isolate Syn7803C15 |
| *Synechococcus* | 147 | KJ019146 | Caudovirales | Myoviridae | Tevenvirinae | *T4likevirus* | ACG-2014f isolate Syn7803C16 |
| *Synechococcus* | 147 | KJ019147 | Caudovirales | Myoviridae | Tevenvirinae | *T4likevirus* | ACG-2014f isolate Syn7803C17 |
| *Synechococcus* | 147 | KJ019148 | Caudovirales | Myoviridae | Tevenvirinae | *T4likevirus* | ACG-2014f isolate Syn7803C19 |
| *Synechococcus* | 147 | KJ019149 | Caudovirales | Myoviridae | Tevenvirinae | *T4likevirus* | ACG-2014f isolate Syn7803C21 |
| *Synechococcus* | 147 | KJ019150 | Caudovirales | Myoviridae | Tevenvirinae | *T4likevirus* | ACG-2014f isolate Syn7803C22 |
| *Synechococcus* | 147 | KJ019151 | Caudovirales | Myoviridae | Tevenvirinae | *T4likevirus* | ACG-2014f isolate Syn7803C24 |
| *Synechococcus* | 147 | KJ019152 | Caudovirales | Myoviridae | Tevenvirinae | *T4likevirus* | ACG-2014f isolate Syn7803C25 |
| *Synechococcus* | 147 | KJ019155 | Caudovirales | Myoviridae | Tevenvirinae | *T4likevirus* | ACG-2014f isolate Syn7803C29 |
| *Synechococcus* | 147 | KJ019159 | Caudovirales | Myoviridae | Tevenvirinae | *T4likevirus* | ACG-2014f isolate Syn7803C34 |
| *Synechococcus* | 147 | KJ019037 | Caudovirales | Myoviridae | Tevenvirinae | *T4likevirus* | ACG-2014f isolate Syn7803C58 |
| *Synechococcus* | 147 | KJ019045 | Caudovirales | Myoviridae | Tevenvirinae | *T4likevirus* | ACG-2014f isolate Syn7803C6 |
| *Synechococcus* | 147 | KJ019052 | Caudovirales | Myoviridae | Tevenvirinae | *T4likevirus* | ACG-2014f isolate Syn7803C7 |
| *Synechococcus* | 147 | KJ019058 | Caudovirales | Myoviridae | Tevenvirinae | *T4likevirus* | ACG-2014f isolate Syn7803C8 |
| *Synechococcus* | 147 | KJ019053 | Caudovirales | Myoviridae | Tevenvirinae | *T4likevirus* | ACG-2014f isolate Syn7803C80 |
| *Synechococcus* | 147 | KJ019066 | Caudovirales | Myoviridae | Tevenvirinae | *T4likevirus* | ACG-2014f isolate Syn7803C9 |
| *Synechococcus* | 147 | KJ019059 | Caudovirales | Myoviridae | Tevenvirinae | *T4likevirus* | ACG-2014f isolate Syn7803C90 |
| *Synechococcus* | 147 | KJ019085 | Caudovirales | Myoviridae | Tevenvirinae | *T4likevirus* | ACG-2014f isolate Syn7803US13 |
| *Synechococcus* | 147 | KJ019086 | Caudovirales | Myoviridae | Tevenvirinae | *T4likevirus* | ACG-2014f isolate Syn7803US17 |
| *Synechococcus* | 147 | KJ019092 | Caudovirales | Myoviridae | Tevenvirinae | *T4likevirus* | ACG-2014f isolate Syn7803US2 |
| *Synechococcus* | 147 | KJ019090 | Caudovirales | Myoviridae | Tevenvirinae | *T4likevirus* | ACG-2014f isolate Syn7803US24 |
| *Synechococcus* | 147 | KJ019091 | Caudovirales | Myoviridae | Tevenvirinae | *T4likevirus* | ACG-2014f isolate Syn7803US26 |
| *Synechococcus* | 147 | KJ019099 | Caudovirales | Myoviridae | Tevenvirinae | *T4likevirus* | ACG-2014f isolate Syn7803US3 |
| *Synechococcus* | 147 | KJ019093 | Caudovirales | Myoviridae | Tevenvirinae | *T4likevirus* | ACG-2014f isolate Syn7803US30 |
| *Synechococcus* | 147 | KJ019095 | Caudovirales | Myoviridae | Tevenvirinae | *T4likevirus* | ACG-2014f isolate Syn7803US34 |
| *Synechococcus* | 147 | KJ019096 | Caudovirales | Myoviridae | Tevenvirinae | *T4likevirus* | ACG-2014f isolate Syn7803US36 |
| *Synechococcus* | 147 | KJ019097 | Caudovirales | Myoviridae | Tevenvirinae | *T4likevirus* | ACG-2014f isolate Syn7803US37 |
| *Synechococcus* | 147 | KJ019098 | Caudovirales | Myoviridae | Tevenvirinae | *T4likevirus* | ACG-2014f isolate Syn7803US39 |
| *Synechococcus* | 147 | KJ019105 | Caudovirales | Myoviridae | Tevenvirinae | *T4likevirus* | ACG-2014f isolate Syn7803US4 |
| *Synechococcus* | 147 | KJ019100 | Caudovirales | Myoviridae | Tevenvirinae | *T4likevirus* | ACG-2014f isolate Syn7803US40 |
| *Synechococcus* | 147 | KJ019101 | Caudovirales | Myoviridae | Tevenvirinae | *T4likevirus* | ACG-2014f isolate Syn7803US42 |
| *Synechococcus* | 147 | KJ019102 | Caudovirales | Myoviridae | Tevenvirinae | *T4likevirus* | ACG-2014f isolate Syn7803US43 |
| *Synechococcus* | 147 | KJ019103 | Caudovirales | Myoviridae | Tevenvirinae | *T4likevirus* | ACG-2014f isolate Syn7803US44 |
| *Synechococcus* | 147 | KJ019106 | Caudovirales | Myoviridae | Tevenvirinae | *T4likevirus* | ACG-2014f isolate Syn7803US50 |
| *Synechococcus* | 147 | KJ019107 | Caudovirales | Myoviridae | Tevenvirinae | *T4likevirus* | ACG-2014f isolate Syn7803US52 |
| *Synechococcus* | 147 | KJ019111 | Caudovirales | Myoviridae | Tevenvirinae | *T4likevirus* | ACG-2014f isolate Syn7803US57 |
| *Synechococcus* | 147 | KJ019123 | Caudovirales | Myoviridae | Tevenvirinae | *T4likevirus* | ACG-2014f isolate Syn7803US7 |
| *Synechococcus* | 147 | KJ019071 | Caudovirales | Myoviridae | Tevenvirinae | *T4likevirus* | ACG-2014g isolate Syn7803US105 |
| *Synechococcus* | 147 | KF156338 | Caudovirales | Myoviridae | Tevenvirinae | *T4likevirus* | ACG-2014h |
| *Synechococcus* | 147 | KJ019082 | Caudovirales | Myoviridae | Tevenvirinae | *T4likevirus* | ACG-2014i isolate Syn7803US120 |
| *Synechococcus* | 147 | KJ019069 | Caudovirales | Myoviridae | Tevenvirinae | *T4likevirus* | ACG-2014j isolate Syn7803US103 |
| *Synechococcus* | 147 | KJ019089 | Caudovirales | Myoviridae | Tevenvirinae | *T4likevirus* | ACG-2014j isolate Syn7803US23 |
| *Synechococcus* | 147 | KC310802 | Caudovirales | Podoviridae | Autographivirinae |  | S-CBP1 |
| *Synechococcus* | 147 | KC310806 |  |  |  |  | S-CBP2 |
| *Synechococcus* | 147 | KC310803 |  |  |  |  | S-CBP3 |
| *Synechococcus* | 147 | KC310804 | Caudovirales | Podoviridae |  |  | S-CBP4 |
| *Synechococcus* | 147 | KC310805 | Caudovirales | Podoviridae |  |  | S-CBP42 |
| *Synechococcus* | 147 | KJ410740 |  |  |  |  | S-EIVl |
| *Synechococcus* | 147 | KF156339 | Caudovirales | Myoviridae | Tevenvirinae | *T4likevirus* | S-MbCM25 |
| *Thalassomonas* | 1 | EU124666.1 | Caudovirales | Podoviridae |  |  | BA3 |
| *Vibrio* | 15 | AB012573.1 |  | Inoviridae |  | *Inovirus* | Vf12 |
| *Vibrio* | 15 | AB012574.1 |  | Inoviridae |  | *Inovirus* | Vf12 |
| *Vibrio* | 15 | AY095314.2 | Caudovirales | Podoviridae |  |  | VpV262 |
| *Vibrio* | 15 | FN297812.1 | Caudovirales | Myoviridae |  |  | VP58.5 |
| *Vibrio* | 15 | HQ316604.1 | Caudovirales | Siphoviridae |  |  | SIO-2 |
| *Vibrio* | 15 | JN849462.1 | Caudovirales | Myoviridae | Tevenvirinae | *Schizot4likevirus* | KVP40 |
| *Vibrio* | 15 | JQ340389.1 | Caudovirales | Siphoviridae |  | *T5likevirus* | pVp-1 |
| *Vibrio* | 15 | JQ692107.1 | Caudovirales | Siphoviridae |  |  | SSP002 |
| *Vibrio* | 15 | JQ801337.1 | Caudovirales | Podoviridae |  |  | VvAW1 |
| *Vibrio* | 15 | JX880072.1 | Caudovirales | Podoviridae |  |  | VPMS1 |
| *Vibrio* | 15 | KC131129.1 | Caudovirales | Myoviridae | Tevenvirinae | *Schizot4likevirus* | VH7D |
| *Vibrio* | 15 | KF800937 |  |  |  |  | AS51 |
| *Vibrio* | 15 | KF322026 | Caudovirales | Podoviridae | Autographivirinae | *T7likevirus* | phi-A318 |
| *Vibrio* | 15 | KM378617 | Caudovirales | Siphoviridae |  |  | VpKK5 |
| *Vibrio* | 15 | KJ936628 |  |  |  |  | VPp1 |
